# Urinary Aminopeptidase Activities as Early and Predictive Biomarkers of Renal Dysfunction in Cisplatin-Treated Rats

**DOI:** 10.1371/journal.pone.0040402

**Published:** 2012-07-05

**Authors:** Andrés Quesada, Félix Vargas, Sebastián Montoro-Molina, Francisco O'Valle, María Dolores Rodríguez-Martínez, Antonio Osuna, Isabel Prieto, Manuel Ramírez, Rosemary Wangensteen

**Affiliations:** 1 Área de Fisiología, Departamento de Ciencias de la Salud, Universidad de Jaén, Jaén, Spain; 2 Departamento de Fisiología, Facultad de Medicina, Universidad de Granada, Granada, Spain; 3 Departamento de Anatomía Patológica, Facultad de Medicina, Universidad de Granada, IBIMER Granada, Spain; 4 Servicio de Nefrología, Hospital Virgen de las Nieves, Granada, Spain; Universidade de Sao Paulo, Brazil

## Abstract

This study analyzes the fluorimetric determination of alanyl- (Ala), glutamyl- (Glu), leucyl-cystinyl- (Cys) and aspartyl-aminopeptidase (AspAp) urinary enzymatic activities as early and predictive biomarkers of renal dysfunction in cisplatin-treated rats. Male Wistar rats (n = 8 each group) received a single subcutaneous injection of either saline or cisplatin 3.5 or 7 mg/kg, and urine samples were taken at 0, 1, 2, 3 and 14 days after treatment. In urine samples we determined Ala, Glu, Cys and AspAp activities, proteinuria, *N*-acetyl-*β*-D-glucosaminidase (NAG), albumin, and neutrophil gelatinase-associated lipocalin (NGAL). Plasma creatinine, creatinine clearance and renal morphological variables were measured at the end of the experiment. CysAp, NAG and albumin were increased 48 hours after treatment in the cisplatin 3.5 mg/kg treated group. At 24 hours, all urinary aminopeptidase activities and albuminuria were significantly increased in the cisplatin 7 mg/kg treated group. Aminopeptidase urinary activities correlated (p<0.011; r^2^>0.259) with plasma creatinine, creatinine clearance and/or kidney weight/body weight ratio at the end of the experiment and they could be considered as predictive biomarkers of renal injury severity. ROC-AUC analysis was made to study their sensitivity and specificity to distinguish between treated and untreated rats at day 1. All aminopeptidase activities showed an AUC>0.633. We conclude that Ala, Cys, Glu and AspAp enzymatic activities are early and predictive urinary biomarkers of the renal dysfunction induced by cisplatin. These determinations can be very useful in the prognostic and diagnostic of renal dysfunction in preclinical research and clinical practice.

## Introduction

Acute kidney injury (AKI) is a common clinical problem that is defined by an abrupt increase in serum creatinine over 48 h resulting from injury or insult that causes a functional or structural change in the kidney. The main cause of AKI is the acute apoptosis or necrosis of renal tubular cells, and the RIFLE (risk, injury, failure, loss, and end-stage renal disease) classification scheme is based in criteria of serum creatinine to classify renal dysfunction in patients [1].

Nevertheless, traditionally used markers of AKI, such as blood urea nitrogen (BUN) and creatinine are insensitive, nonspecific, and do not adequately differentiate between the different stages of AKI [2]. Serum creatinine accumulates over time, and changes in creatinine concentrations become apparent only when the kidneys have lost 50% of their functional capacity [3], [4].

Early detection of AKI remains a challenge in both preclinical research and clinical practice. There is an urgent need for better biomarkers to permit more timely diagnosis of AKI, prediction of injury severity, and safety assessment during drug development [2].

Enzymes released from damaged tubular cells and excreted into urine are the most promising biomarkers for an early detection of AKI. They have an obvious diagnostic benefit because their measurements may provide detailed information about the nature, size and site of the damage to tubular cells and their possible necrosis or dysfunction [5]. One of these enzymes, AlaAp (EC 3.4.11.2), is an enzyme of the brush border proposed in the early seventies as a urinary marker of renal disease [6], and there is an automatic photometric assay for its determination [7]. AlaAp, together with GluAp (EC 3.4.11.7), CysAp (3.4.11.3) and AspAp (EC 3.4.11.21) are present in the renal tubular cells [8]–[10] and they have an aminopeptidasic function in angiotensin II metabolism, peptide that is increased in renal diseases. In our laboratory, we have recently determined the activity of these four aminopeptidases as an index of renal damage in salt-treated and hyperthyroid rats [11].

In this work we investigated the reliability of the fluorimetric determination of AlaAp, GluAp, CysAp and AspAp urinary enzymatic activities as biomarkers of renal dysfunction induced by cisplatin at two different doses (3.5 and 7 mg/kg) in rats. Cisplatin is an antineoplastic drug known for its direct proximal tubular nephrotoxicity in both humans and animals [12], [13]. Rats treated with one single dose of cisplatin at 7 mg/kg exhibit tubular degeneration and necrosis [14] accompanied with a maximal increase in the excretion of NAG [15], a renal tubular enzyme [16], and glucosuria [15] at the third day of injection. At day 7, plasma creatinine and BUN reach a maximum [15] and tubular epithelial regeneration and dilatation are observed on day 8 after treatment [14]. From day 14 to 56, development of tubulointersticial fibrosis is observed in these rats with no differences in urinary enzymes or glucose, but a significant increase in plasma creatinine and BUN at day 56 [15].

We studied aminopeptidase activities as early biomarkers of AKI and their ability to detect a slight renal damage evaluating their increased urinary excretion at the first three days from injection. We also analyzed their value as predictive biomarkers of the severity of renal dysfunction by correlating the first day urinary excretion level of the marker with two parameters of renal function (plasma creatinine and creatinine clearance) and two parameters of structural damage (renal hypertrophy and interstitial fibrosis). We determined other parameters commonly used as urinary biomarkers of renal damage (proteinuria, NAG, albumin, and NGAL) and made combined ROC area under the curve (AUC) analysis in order to establish their sensitivity and specificity to detect renal alterations.

## Materials and Methods

### Ethics statement

All experimental procedures were performed according to the European Union Guidelines to the Care and Use of Laboratory Animals and approved by the Ethical Committee of the University of Jaén with the approval ID R1/12/2010/66.

### Animals and drugs

24 male Wistar rats weighing 227–279 g were purchased from Harlan Laboratories (Barcelona, Spain). These rats were kept in a room maintained at 24±1°C and humidity of 55±10%, with a 12-h light/dark cycle and had free access to rat chow and tap water. Cisplatin (Sigma, Madrid, Spain) was dissolved in saline (1.5 and 3 mg/ml).

### Experimental protocols

In order to examine the time course enzymatic activities, rats were distributed in three groups: Control, CisPt3.5 and CisPt7, (n = 8 each group), that received a subcutaneous injection of either saline or cisplatin 3.5 mg/kg and 7 mg/kg, respectively. One day before treatment and at 0, 1, 2 and 13 days after cisplatin or saline administration, rats were housed in metabolic cages and 24-h urine collection was made, obtaining urine samples at 0, 1, 2, 3 and 14 days. Urine samples were centrifuged 15 min at 1000 g, aliquoted and frozen at −80°C until assay. At the end of the experiment, blood samples were obtained from left ventricle under anesthesia (pentobarbital, 50 mg/kg, i.p.), centrifuged 15 min at 1000 *g*, aliquoted and stored at −80°C. Kidneys were removed and weighted. One kidney was fixed in 10% neutral-buffered formalin solution during 24 h and subsequently placed in 70% ethanol.

### Analytical procedures

Ala, Glu, Cys and AspAp urinary activities were determined in duplicate in a kinetic fluorimetric assay using alanyl-, glutamyl-, cystinyl- and aspartyl-β-naphtylamide as substrates, respectively. 20 µl of urine were incubated during 30 min at 37°C with 90 µl of their corresponding substrate solution (2.14 mg/dl alanyl-β-naphtylamide, 10 mg/dl bovine serum albumin (BSA), 10 mg/dl dithiotreitol (DTT) in pH 7.4 50 mM HCl-Tris; 2.72 mg/dl glutamyl-β-naphtylamide, 10 mg/dl BSA, 10 mg/dl DTT and 555 mg/dl CaCl_2_ in pH 7.4 50 mM HCl-Tris; 5.63 mg/dl cystinyl-β-naphtylamide, 10 mg/dl BSA, 10 mg/dl DTT in pH 6 50 mM HCl-Tris; 2.58 mg/dl aspartyl-β-naphtylamide, 10 mg/dl BSA, 555 mg/dl CaCl_2_ in pH 7.4 50 mM HCl-Tris). The substrates were previously dissolved in 1 ml of dimethyl sulfoxide and stored at −20°C. The amount of β-naphtylamine released as a result of the aminopeptidase activities was measured fluorimetrically at an emission wavelength of 412 nm with an excitation wavelength of 345 nm, and quantified using a standard curve of β-naphtylamine (0–200 nmol/ml). Sample blanks were made in duplicate using an incubation solution that did not contain the substrate of the enzyme. Fluorimetric data from samples, blanks and standard curve were taken each minute, and fluorescence of sample blanks was subtracted from the fluorescence of the samples at each point. Specific aminopeptidase activities were calculated from the slope of the linear portion of enzymatic assay obtaining nanomol of substrate hydrolyzed per ml and minute. These values were normalized by diuresis and expressed as absolute excretion per 100 g of rat and day.

Proteinuria was determined with the red pirogallol protein assay (Spinreact, Barcelona, Spain). NAG was determined by a colorimetric method purchased from Roche Diagnostics (Barcelona, Spain). Urinary albumin and NGAL were analyzed by ELISA with kits purchased from Bethyl Laboratories (Montgomery, TX, USA) and Bioporto Diagnostics (Gentofte, Denmark), respectively. Plasma and urinary creatinine was measured by the kinetic method of Jaffé, based on the reaction of creatinine with sodium picrate.

These urinary parameters were measured at 0, 1, 2 and 3 days from injection of cisplatin and expressed as absolute excretion per 100 g of rat and day. Plasma and urinary creatinine concentration were measured at the end of the experiment.

### Histopathological analysis

For conventional morphology, buffered 10% formaldehyde-fixed, paraffin-embedded transversal kidney sections in horizontal plane were stained with hematoxylin and eosin, Masson's trichrome and periodic acid-Schiff stain (PAS). The morphological study was done in blinded fashion on 4-micrometer sections with light microscopy, using the most appropriate stain for each lesion. The severity of lesions was calculated semiquantitatively using a 0 to 3 scale (0, absence; 1, mild [<10% of juxtamedullary proximal tubules, vessels or glomeruli involved]; 2, moderate [10 to 25%]; 3, severe [>25%]).

### Morphometrical analysis

Kidney samples fixed in buffered 10% formalin were embedded in paraffin and serially sectioned at 5 µm thickness. Afterwards, they were stained with 1% picro Sirius red F3BA (Gurr, BDH Chemicals Ltd., Poole, United Kingdom) for image analysis quantification. To improve staining, tissue sections were kept after deparaffination for 3–5 days in 70% ethanol as mordent. Picro Sirius red stains connective fibers deep red and cell nuclei and cytoplasmatic structures light red/bright yellow [17].

To automatically quantify interstitial connective tissue and glomerular morphometry on rats kidney histologic sections, we developed several image processing algorithms that had been brought together in one image analysis application, named Fibrosis HR® [18]. We evaluated 20 images of corticomedullary junction per kidney as previously described [18].

### Statistical analyses

We used *t* test for the analysis of variables with normal distribution. Mann-Whitney W (Wilcoxon) test was used to analyze the differences in morphological variables between treated and control rats. Differences were considered statistically significant at P<0.05 level. Linear regression and analysis of variance were made to establish the correlation of urinary biomarkers at the first day with plasma creatinine, creatinine clearance, renal hypertrophy and interstitial fibrosis at the end of the experiment. P<0.05 and |r| >0.5 was considered as a strong correlation. ROC-AUC analysis was made with JLABROC4 software [19]. We used the results obtained at 24 hours of treatment, and studied the sensitivity and specificity of each marker to differentiate if a rat had received cisplatin (either 3.5 or 7 mg/kg) or saline.

## Results

**Figure 1:**
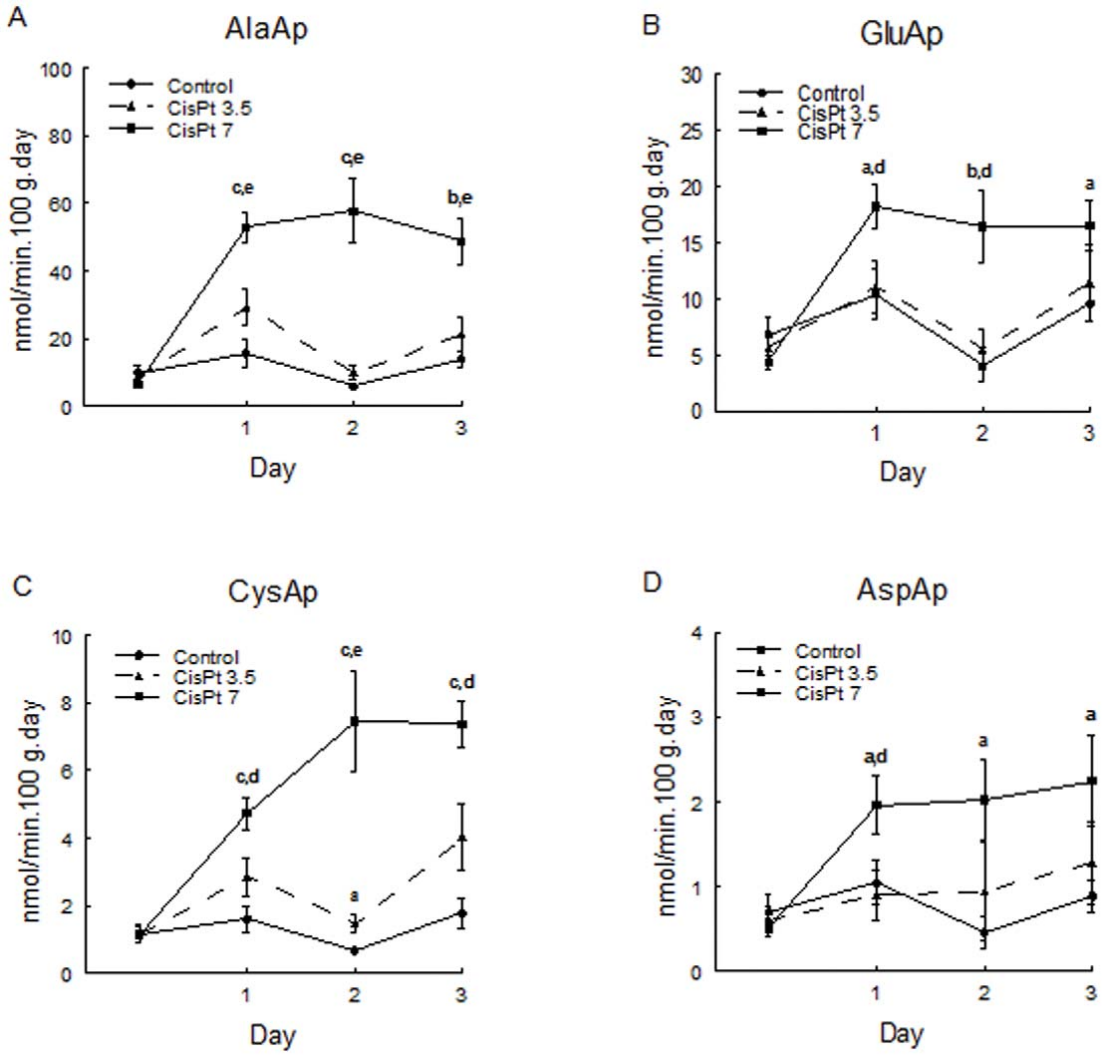
Urinary aminopeptidase activities. AlaAp (A), GluAp (B), CysAp (C), and AspAp (D) urinary activities excreted per day and 100 g of rat at 0, 1, 2 and 3 days of treatment in control, CisPt3.5 and CisPt7 groups. Data are means ± SEM. **a** p<0.05, **b** p<0.01, **c** p<0.001 compared with control group. **d** p<0.05, **e** p<0.01 compared with CisPt3.5 group.

Figure 1 shows the time course of aminopeptidase urinary excretion in experimental groups. Ala, Glu, Cys and AspAp activities were increased at day 1, 2 and 3 after treatment in CisPt7 group. We also found a significantly increase of CysAp activity at day 2 in CisPt3.5 group (Fig. 1C).

**Figure 2:**
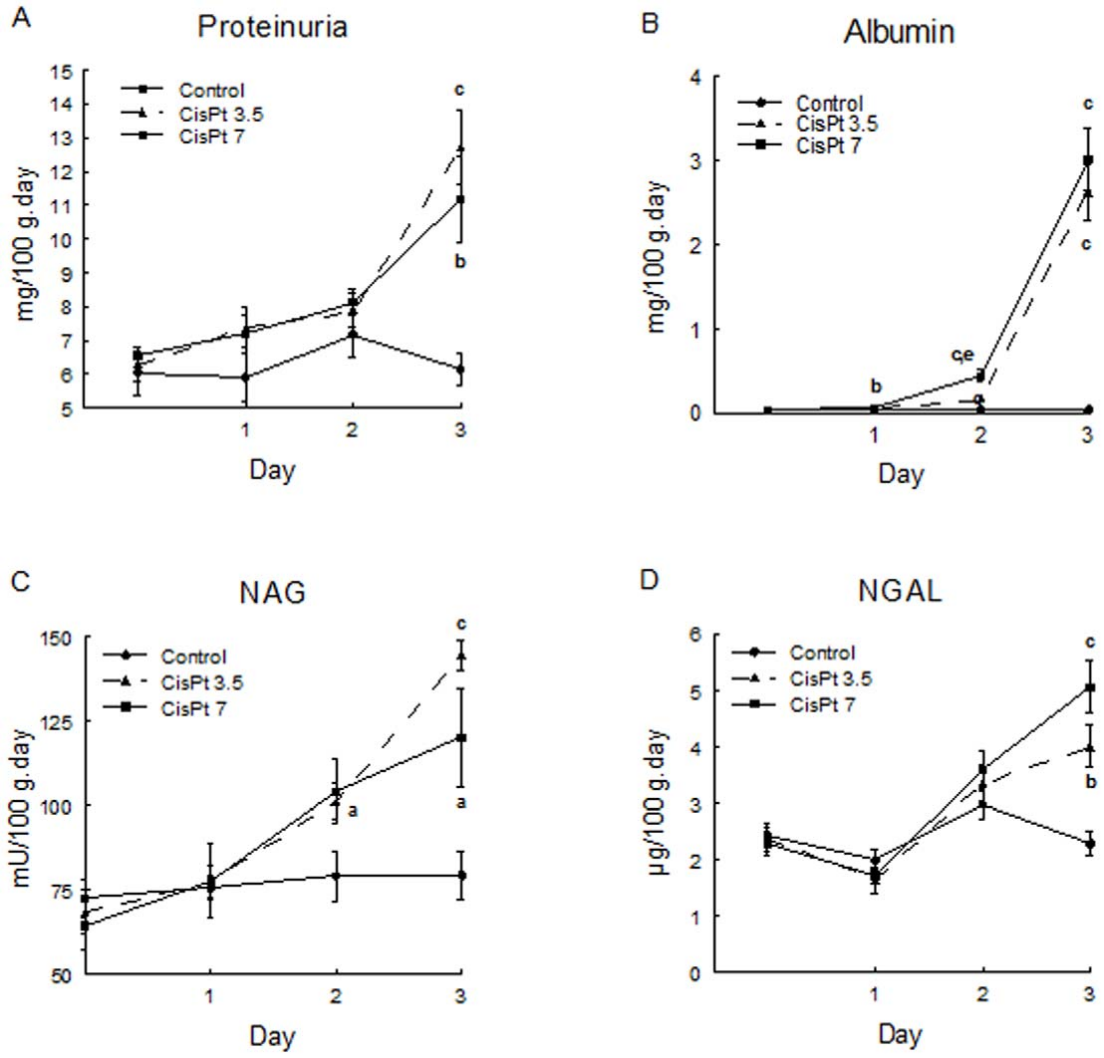
Proteinuria, albuminuria, NAG and NGAL excretion. Proteinuria (A), albuminuria (B), *N*-acetyl-*β*-D-glucosaminidase (C) and neutrophil gelatinase-associated lipocalin (NGAL) excreted per day and 100 g of rat at 0, 1, 2 and 3 days of treatment in control, CisPt3.5 and CisPt7 groups. Data are means ± SEM. **a** p<0.05, **b** p<0.01, **c** p<0.001 compared with control group. **d** p<0.05, **e** p<0.01 compared with CisPt3.5 group.

Albuminuria was increased at day 1, 2 and 3 in CisPt7 group, while proteinuria, NAG and NGAL were increased at day 3 (Fig. 2). Albuminuria and NAG increased at day 2 and 3 in CisPt 3.5 group. Proteinuria and NGAL excretion were also significatively augmented at day 3 in CisPt 3.5 group.

**Figure 3:**
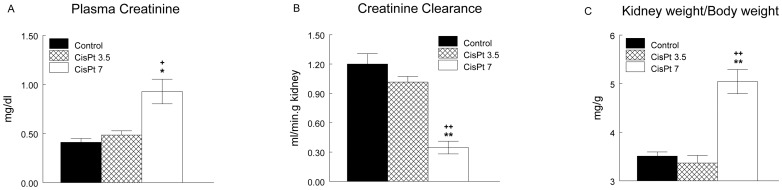
Plasma creatinine, creatinine clearance and kidney weight/body weight ratio at the end of the experiment. Plasma creatinine (A), creatinine clearance (B) and kidney weight/body weight ratio (C) at the end of the experiment in control, Cispt3.5 and CisPt7 groups. *p<0.01, **p<0.001 compared with control group. + p<0.01, ++ p<0.001 compared with CisPt3.5 group.

At the end of the experiment, CisPt7 group exhibited an augmented plasma creatinine level, diminished creatinine clearance and renal hypertrophy (Fig. 3), while CisPt3.5 group did not show any statistically significative difference in these parameters with respect to control group.

**Figure 4:**
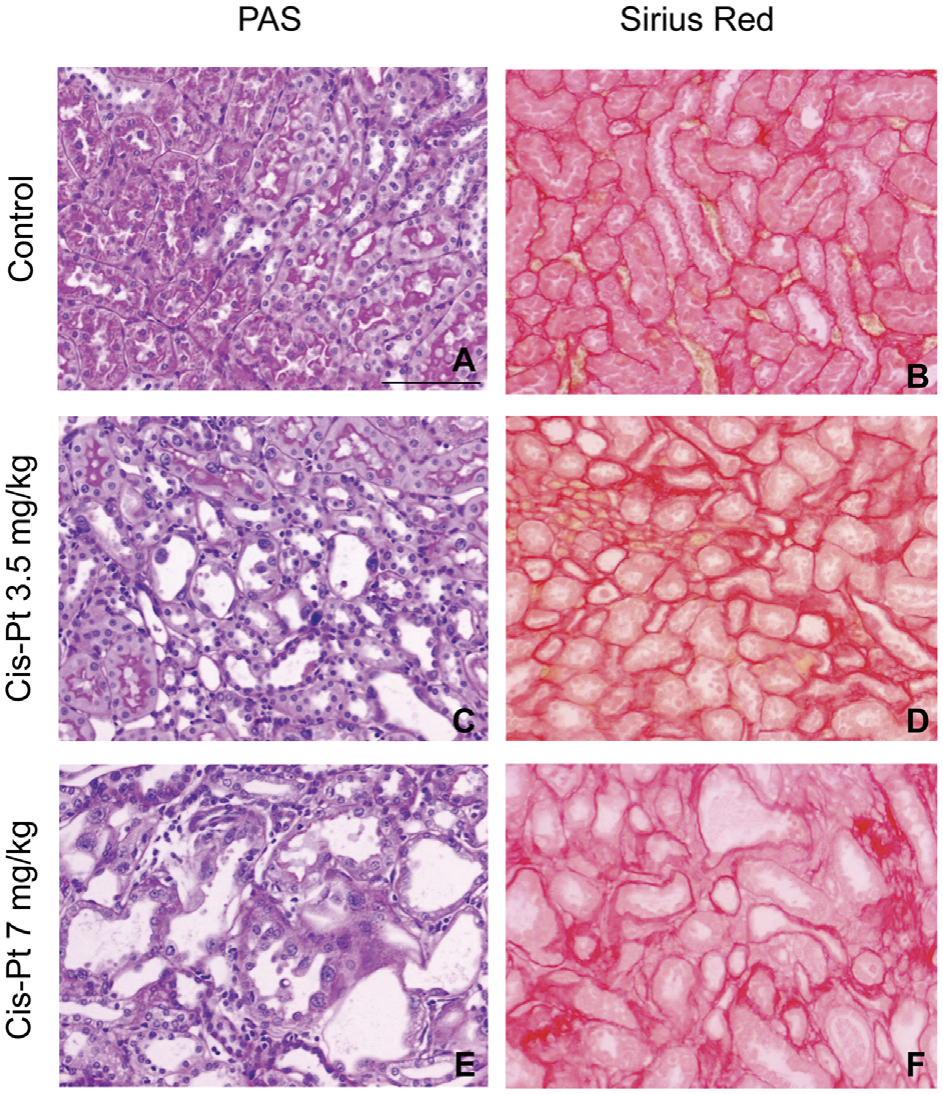
Morphological changes and tubulointerstitial fibrosis in renal outer medulla induced by cisplatin. Left panel shows control group without morphological lesion (A), CisPt3.5 (C) and CisPt7 (E) groups whit moderate nuclear dysplasia, incipient acute tubular necrosis, marked tubular dilation and apoptotic cells in tubular lumen of proximal convoluted tubules in the corticomedullary junction (PAS original magnification x200). Scale bar  = 100 micrometers. Right panel shows representative photographs of Sirius red staining of kidneys from control (B), 3.5 (D) and 7 mg/kg cisplatin-treated rats (F) after 14 days (original magnification x200).

The renal lesions in Wistar control rats were absent. No glomerular, tubulointerstitial or vascular lesions were present in renal parenchyma. Histopathological examination of renal slices from rats treated with 3.5 or 7 mg/kg of cisplatin showed different alterations, including relevant nuclear dysplasia and incipient acute tubular necrosis (Fig. 4). The sections of the kidney from cisplatin-treated rats exhibited marked dilation of proximal convoluted tubules in the corticomedullary junction with slogging of almost entire epithelium due to desquamation of tubular epithelium that induced a total absence of microvilli and loss of brush border in CisPt7 group. Also, mild tubular atrophy and apoptotic cells in tubular lumen were present in both cisplatin-treated groups (Table 1). Statistical differences were found between both cisplatin groups into morphological variables analysis, indicating a dose-response relationship.

**Table 1:** Morphological and morphometrical variables analyzed in kidney biopsies from Control, CisPt3.5 and CisPt7 rats at the end of the experiment.

Groups	Control	CisPt 3.5	CisPt 7
Displasia S3 segment	0.00 ± 0.00	1.63 ± 0.18**	2.75 ± 0.16**^,++^
Acute tubular necrosis	0.00 ± 0.00	1.13 ± 0.23*	2.13 ± 0.13**^,+^
Apoptosis	0.00 ± 0.00	1.25 ± 0.25*	2 ± 0.27**
Tubular casts	0.00 ± 0.0 0	0.13 ± 0.13	1.38 ± 0.26* ^,+^
Tubular atrophy	0.00 ± 0.00	0.00 ± 0.00	0.13 ± 0.13
Tubular dilation	0.00 ± 0.00	1.00 ± 0.19*	2.75 ± 0.16**^,++^
Tubular vacuolization	0.00 ± 0.00	0.00 ± 0.00	0.00 ± 0.00
Tubular mitosis	0.00 ± 0.00	0.50 ± 0.19	1.25 ± 0.16**^,+^
Inflammatory infiltrate	0.00 ± 0.00	0.20 ± 0.20	0.50 ± 0.33
Glomerular lesion	0.00 ± 0.00	0.00 ± 0.00	0.00 ± 0.00
Vascular lesion	0.00 ± 0.00	0.00 ± 0.00	0.20 ± 0.20
Fibrosis (%)	2.61 ± 0.28	5.28 ± 0.43**	5.36 ± 0.50**
Fibrosis area (µm^2^)	3591 ± 388	7194 ± 548**	7361 ± 660**

* p<0.01, **p<0.001 *versus* control group. +p<0.01, ++p<0.001 *versus* Cispt3.5 group. n = 8 each group.

Cisplatin significantly enlarged the fibrosis area in the tubular interstitium from CisPt3.5 and CisPt7 groups. The fibrosis area was remarkably higher in the corticomedullary junction of the kidney (Table 1, Fig. 4).

**Figure 5:**
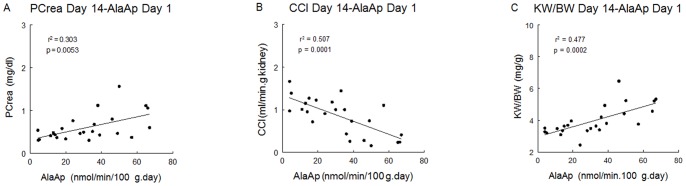
Linear regressions between urinary AlaAp activity at day 1 and plasma creatinine, creatinine clearance and kidney weight/body weight at day 14. Linear regressions between AlaAp activity at day 1 and plasma creatinine (A), creatinine clearance (B) and kidney weight/body weight (C) at the end of the experiment.

Significative correlations were found for Ala, Glu, Cys and AspAp urinary activities at 24 hours of treatment with kidney weight/body weight ratio at the end of the experiment. AlaAp and CysAp activities correlated with creatinine clearance, and AlaAp also correlated with plasma creatinine (Table 2, Fig. 5). We did not found a correlation of any of biomarkers studied at this point with renal fibrosis (Table 2).

**Table 2:** P-value and correlation coefficients (r and r^2^) of linear regression between urinary biomarkers excreted at day 1 *versus* kidney weight/body weight ratio, interstitial fibrosis, plasma creatinine and creatinine clearance at day 14.

	P value	R	R^2^
*Kidney weight/body weight*			
AlaAp*	0.0002	0.690	0.477
CysAp*	0.0009	0.632	0.400
GluAp*	0.0040	0.565	0.319
AspAp*	0.0110	0.509	0.259
NGAL	0.2877	−0.226	0.051
Proteinuria	0.4219	0.172	0.030
NAG	0.7564	0.067	0.447
Albumin	0.7056	0.081	0.007
*Interstitial fibrosis*			
AlaAp	0.0621	0.386	0.149
Albumin	0.1473	0.305	0.093
CysAp	0.2410	0.249	0.062
AspAp	0.3673	0.193	0.037
GluAp	0.3843	0.186	0.035
NGAL	0.6847	−0.087	0.008
Proteinuria	0.8324	0.046	0.002
NAG	0.9255	−0.020	0.000
*Plasma creatinine*			
AlaAp*	0.0053	0.550	0.303
CysAp	0.0561	0.395	0.156
Albumin	0.0607	0.388	0.151
AspAp	0.1650	0.293	0.086
GluAp	0.2437	0.247	0.061
NAG	0.2511	−0.244	0.059
Proteinuria	0.8290	−0.047	0.217
NGAL	0.8905	−0.030	0.088
*Creatinine clearance*			
AlaAp*	0.0001	−0.712	0.507
CysAp*	0.0015	−0.611	0.374
GluAp	0.0142	−0.494	0.244
AspAp	0.0158	−0.487	0.237
Albumin	0.1036	−0.340	0.116
Proteinuria	0.4234	−0.174	0.029
NGAL	0.7485	0.069	0.005
NAG	0.9409	0.016	0.000

* p<0.05 and |r| >0.5.

Biomarkers are rank ordered for p-value from top to bottom.

**Figure 6:**
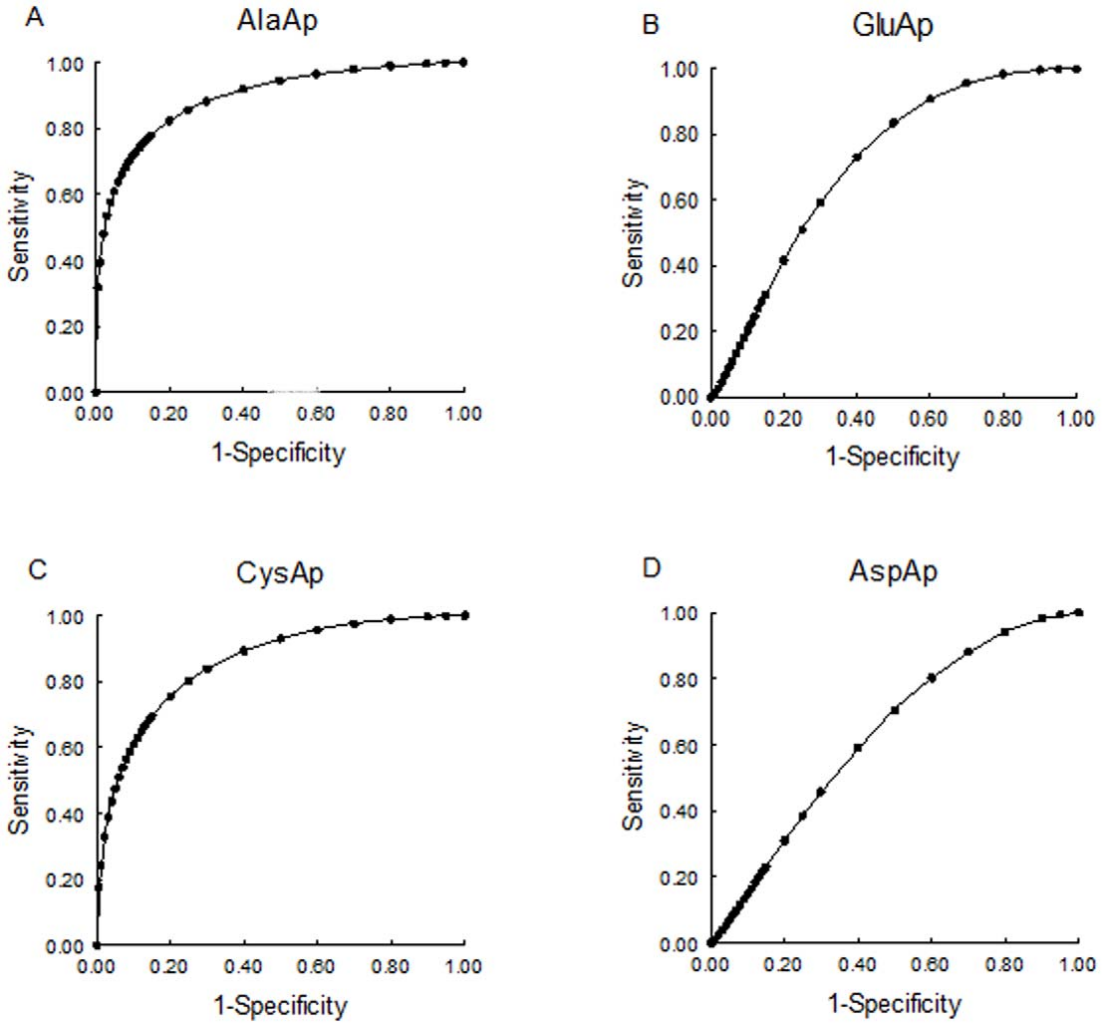
ROC curves for urinary aminopeptidase activities at day 1. ROC curves showing specificity and sensitivity for AlaAp (A), GluAp (B), CysAp (C) and AspAp (D) urinary activities at the first day of treatment.

AlaAp presented the largest ROC-AUC of all the markers studied 24 hours after treatment (Fig. 6). Therefore, AlaAp showed the maximum levels of specificity and sensitivity to detect if an animal received cisplatin or saline and it was a perfect discriminator between treated and untreated rats. GluAp, CysAp and AspAp also had an AUC >0.5, as albuminuria and proteinuria (Table 3), indicating that these enzymes could also be considerated as biomarkers of renal dysfunction in this animal model.

**Table 3:** ROC-Area under the curve (AUC), sensitivity at 95% specificity (Sens 95%) and fold-cutoff relative to controls to achieve 95% specificity (threshold) of urinary biomarkers excreted at day 1 in control (true negative) and cisplatin 3.5 or 7 mg/kg (true positive) treated rats.

	AUC	Sens 95%	Threshold
AlaAp	0.894±0.067	0.6090	1.7394
CysAp	0.860±0.079	0.4757	1.6209
Albumin	0.806±0.087	0.5321	1.7196
GluAp	0.714±0.119	0.0895	1.7079
Proteinuria	0.709±0.114	0.1354	1.6543
AspAp	0.633±0.125	0.0683	1.6299
NAG	0.453±0.114	0.1492	1.5392
NGAL	0.377±0.124	0.0129	1.7247

Biomarkers are rank ordered for AUC from top to bottom.

## Discussion

The main finding of this article is that Ala, Glu, Cys, and AspAp activities are early and predictive urinary biomarkers of the AKI induced by cisplatin, and their determination could be very useful in early detection and monitorization of renal damage.

Biochemical and histhopathological data obtained in our experimental groups were in consonance with those obtained in previous works by other authors [14], [15] and confirmed the effectiveness of cisplatin treatment producing renal injury, showing similar increases in plasmatic creatinine, proteinuria and enzymuria at the same days of the study.

Ala, Glu and CysAp are highly organ-specific because they are present in the brush border membrane of renal tubular cells [9] and exhibit high molecular weights (>140 kDa) that make difficult that their presence in urine could be due to alterations in glomerular barrier. These enzymes participate in the intrarrenal renin-angiotensin-aldosterone system (RAAS) [20]. It is known that increased tubular absorption of filtered proteins induces tubulointerstitial inflammation, ultimately resulting in tubular atrophy, interstitial fibrosis, and loss of renal function [21]. In the proximal tubule, albumin and other ultrafiltered proteins are reabsorbed by endocytosis involving megalin and cubulin [22]. AngII stimulates albumin endocytosis in proximal tubule cells *via* AT2 receptor–mediated protein kinase B activation. However, an increase in tubular albumin reabsorption activates the tubular RAAS, leading to a vicious circle [23] that could explain the elevated urinary activities of these enzymes when they are released from the brush border to the ultrafiltrate after cisplatin injection.

In consequence, AlaAp activity has been widely used as a marker of renal dysfunction in animal models of nephrotoxicity induced with vancomicine [24] or amphotericin B [25], or in human diseases like glomerulopathies [26], IgA nephropathy [27], rheumatoid arthritis [28] or diabetes [29], and there are contradictory results about its utility as a marker in kidney transplanted patients [30], [31].

The high correlation of urinary activities of Glu, Ala, Cys and AspAp at day 1 with kidney weight/body weight ratio, and of Ala and CysAp with creatinine clearance at day 14 shows their predictive value over renal dysfunction and structural damage. AlaAp also correlates with plasma creatinine at day 14. Nevertheless, there is no correlation in any of the biomarkers studied with renal fibrosis. Kawai et al [15] concluded that fibrosis is a process of resolution from acute tubular injury induced by cisplatin and, in our study, we also have found an increase in the area of interstitial fibrosis at day 14 in the two groups of animals treated with cisplatin. The lack of correlation of all urinary biomarkers with renal fibrosis may indicate that the extent of fibrotic lesions is not directly related with the tubular injury evoked by cisplatin at 24 hours. In fact, the group treated with the submaximal dose of cisplatin showed a fibrosis area similar to the group treated with 7 mg/kg. Nevertheless, the CisPt3.5 group had unaltered kidney weight/body weight, plasma creatinine or creatinine clearance. This would indicate that the alterations in renal function observed in CisPt7 group are more dependent of other factors like tubular dilation or dysplasia rather than of the fibrotic process.

Interestingly, the rest of the markers studied did not correlate with any parameter of renal dysfunction or structural damage, and albuminuria was the only marker that was slightly increased at 24 hours in CisPt7 group. Low-molecular weight proteins like albumin can pass through glomerular barrier and, therefore, urinary proteins and albumin may have an extrarrenal origin in other patologies. In a previous study, albuminuria has been related with cardiovascular events like endothelial injury that presents collateral kidney damage [32]. Therefore, the urinary levels of albumin or total proteins may be influenced by their plasmatic concentration, making difficult to elucidate in some cases if their presence in urine is exclusively due to the renal alterations. In our experiment, the slight elevation of albuminuria at 24 hours in CisPt7 group may be probably related with the reduction in the tubular reabsorption of this protein due to the release of aminopeptidases from microvilli, because these enzymes were the only markers that were increased at this point.

24 hours after treatment there were no differences in the excretion of NAG and NGAL in the animals treated with the submaximal or the maximal dose of cisplatin *versus* control group. Therefore, in this model, NAG and NGAL could not detect the renal damage evoked with cisplatin as early as aminopeptidase activities or albuminuria and they do not predict renal alterations 24 hours after treatment as Ala, Glu, Cys or AspAp activities do. NAG and NGAL are implicated in several tubular functions that include transport of proteins [16] and hydrophobic molecules [33] through membranes, respectively. Our findings suggest that the release of aminopeptidases from brush border precedes in time to the alterations in these renal tubular functions in this model. NAG and albuminuria started to increase at day 2 in CisPt3.5 group, and NGAL and proteinuria at day 3, while aminopeptidase activities were not significatively increased, except a slight increase in CysAp activity at day 2. This would indicate that the submaximal dose of cisplatin evokes alterations in renal tubular transport functions that are similar to the alterations observed with the maximal dose of cisplatin. In fact, there were no differences in the excretion of protein, albumin, NAG and NGAL between CisPt3.5 and CisPt7 group. Nevertheless, this tubular dysfunction is not accompanied with a significative loss of brush border enzymes or, at least, it is not sufficiently remarkable with this submaximal dose.

In conclusion, Ala, Glu, Cys and AspAp enzymatic activities are early and predictive biomarkers of the AKI induced by cisplatin. The four markers show high sensitivity and specificity to distinguish nephrotoxicant-treated from control rats. These determinations could be very useful in the prognostic and diagnostic of renal dysfunction in preclinical research and clinical practice, because the urine sample does not need any special treatment and the laboratory test is simple, quick and inexpensive in comparison with other techniques that require expensive antibodies and large periods of incubation, like ELISA or Western blot, that sometimes make difficult their serial determination.
